# WaveUNet+: Preserving Root System Architecture Integrity in In Situ Root Segmentation via a Unified Spectral–Spatial Framework

**DOI:** 10.3390/plants15132034

**Published:** 2026-06-30

**Authors:** Liuli Wang, Meng Zhang, Xingyun Liu, Qiushi Yu, Lingxiao Zhu, Liantao Liu, Nan Wang

**Affiliations:** 1 State Key Laboratory of North China Crop Improvement and Regulation, Hebei Agricultural University, Baoding 071000, China; 15603219764@163.com (L.W.); zhangmeng8426@163.com (M.Z.); a820919625@163.com (X.L.); jiwd123456@gmail.com (Q.Y.); zlxhbnydx@163.com (L.Z.); liultday@126.com (L.L.); 2 College of Mechanical and Electrical Engineering, Hebei Agricultural University, Baoding 071000, China; 3 College of Agronomy, Hebei Agricultural University, Baoding 071000, China

**Keywords:** In Situ roots, wavelet transform, U-Net3plus, Docker

## Abstract

Root phenotypic analysis is closely related to crop yield and stress resistance. Although deep learning can improve the efficiency of root phenotype recognition, existing methods suffer from insufficient segmentation accuracy under complex soil backgrounds and focus on a single target. To address the issues of limited accuracy and operational complexity in existing root segmentation models, this paper proposes a novel wavelet-enhanced full-scale segmentation network. The WaveUNet+ model is based on U-Net3plus, replaces traditional downsampling with the Haar wavelet transform, and introduces the EMA module. The impact of the wavelet transform is validated using Grad-CAM, and HD95 is employed to evaluate the improvement in segmentation quality brought by the attention mechanism from the perspective of boundary accuracy. Transfer learning is used to improve model generalization, and the test results on diverse roots and various soils are compared. A Docker containerized root image segmentation method is designed to achieve convenient and practical operation, and the deployment feasibility of the model on edge devices is also verified. Our model effectively enhances the recognition of fine roots in soil backgrounds, leading to improvements across various metrics, achieving an Accuracy of 99.2%, while improving model accuracy with relatively low parameter count and model size. Compared with the original U-Net model, mIoU is increased by 1.52% and Recall by 2.93%. The results show that the model not only performs excellently on the original dataset but also maintains good generalization ability across different imaging modalities, crop species, and soil conditions. With Docker, users can achieve root image segmentation on their own computers without tedious program installation and environment configuration. In the future, we will attempt methods such as pruning and quantization to reduce model size, so as to better adapt to the deployment requirements of edge devices.

## 1. Introduction

Root systems are the primary organs through which plants absorb water and nutrients [1]. Studying root systems can reveal how plants adapt to conditions such as drought, waterlogging, salinity, and low temperatures through changes in root morphology, architecture, and physiological traits [2]. In agricultural production, robust root development forms the foundation for high crop yields. Root phenotypic analysis is closely linked to crop yield and stress resistance. Through precise phenotypic analysis, superior varieties with well-developed root systems and strong stress resistance can be screened, providing key indicators for crop breeding. This accelerates the breeding process and facilitates the development of crop varieties better adapted to complex environments and high-yield demands [3].

Traditional root research methods, such as excavation, soil coring, container-based methods, and root-box techniques, often cause significant damage to roots and struggle to simulate natural soil growth conditions. With technological advancements, root research methodologies have improved accordingly [4,5,6]. Commonly used in situ imaging approaches include minirhizotron systems [7], hyperspectral imaging [8], X-ray scanning technology [9,10], 3D magnetic resonance imaging, and digital imaging devices [11,12,13]. Minirhizotron camera systems can capture time-series images of roots to record changes in individual roots, such as birth, death, and elongation. However, the installation of minirhizotron tubes requires drilling into the soil, which may sever or damage part of the root system. The diameter and length of minirhizotron tubes are usually limited, generally allowing observation only of roots within a small radius around the tube. For plants with widely distributed or complex root architectures, important root information may be missed. HSI (Hyperspectral imaging), as a non-destructive technique, enables comprehensive and in-depth analysis and dynamic monitoring of plant tissue and organ development. Unfortunately, applications of HSI to roots and the root–soil interface remain relatively scarce, and the requirements for data processing and analysis are high. X-ray technology [14] and 3D magnetic resonance imaging allow in situ observation of roots without damaging the plant root system or the surrounding soil environment. Their drawbacks include relatively long imaging times, expensive equipment, and high maintenance costs. The RhizoPot platform, developed earlier in our laboratory, enables non-destructive acquisition of complete root images using a scanner combined with the RhizoPot growth device [15]. In this study, the RhizoPot system was selected to collect in situ root images as the dataset.

Root segmentation is the foundation of phenotypic analysis. Traditional root analysis methods often rely on manual measurement and observation, which are not only time-consuming and labor-intensive but also limited in accuracy. Advances in deep learning have opened up new possibilities for automated and precise root segmentation [16,17,18]. U-Net, a commonly used segmentation model, was initially applied in the medical field [19]. The U-Net3plus model incorporates full-scale skip connections, fusing feature maps from different scales of the encoder with corresponding layers in the decoder, leading to more accurate segmentation of fine and complex structures [20]. Other widely used segmentation networks include SegNet [21], FCN [22], and U-Net2plus [23]. These models typically employ classical classification networks such as ResNet [24] and VGG [25] as backbone encoders. In response to the complexity and adaptability required for lung tumor image segmentation, a parallel deep-learning image segmentation algorithm with a hybrid attention mechanism has been proposed [26]. In the context of plant root segmentation [27], the DeepLabv3+ semantic segmentation model has been enhanced by integrating sub-pixel convolution and further incorporating attention mechanisms, yielding improved segmentation performance [28]. Tang et al. [29] introduced a transformer-based neural network technique for retrieving in situ root senescence characteristics in cotton (*Gossypium hirsutum* L.). Attention mechanisms can enhance the performance of segmentation network models by focusing on key regions of the image, thereby improving the model’s ability to handle details and contextual information [30]. With the continuous development of computer vision and deep learning technologies [31], deep-learning-based plant root segmentation has become a prominent research focus [32].

Convolution serves as the core building block of convolutional neural networks, and its feature fusion mechanism lays a crucial foundation for the precise implementation of image segmentation tasks. Shelhamer et al. [22] overcame the limitation of fixed input sizes by replacing fully connected layers in traditional classification networks with convolutional layers, enabling end-to-end pixel-wise prediction. Hu et al. [33] further clarified the role of convolution in segmentation networks by decomposing convolutional segmentation networks into category-aware convolutional kernels, demonstrating that such kernels can capture feature information specific to particular categories. MSC-Net combines a convolutional backbone with a multi-scale self-attention module, improving multi-scale feature fusion and boundary quality for semantic segmentation while maintaining computational efficiency [34]. Per-SAM-MCPA achieves fine-grained segmentation in high-density scenes by integrating multi-head cross parallel attention and a compound constraint loss function, balancing region completeness and boundary accuracy [35].

Integrating wavelet transforms into CNN architectures, Haar wavelet transform is applied to reduce the spatial resolution of feature maps while preserving as much information as possible [36]. Incorporating Discrete Wavelet Transform into U-Net for blurry concrete crack images has been shown to enhance the performance of semantic segmentation models [37]. Agnes et al. [38] demonstrated the effectiveness of combining wavelet transforms with U-Net++ for accurate lung nodule segmentation. Wavelet transforms decompose signals into different frequency channels and scales, which aids in capturing features at multiple levels [39,40]. Current deep learning-based methods for plant root segmentation still have several problems [41]. The grayscale difference between fine roots and soil background is subtle, and existing methods are prone to losing high-frequency edge information of these fine structures during downsampling, causing fine roots to be misclassified as background and resulting in a high missed-detection rate. In areas where soil color is very close to that of the roots, current models struggle to precisely locate root boundaries, leading to shifted segmentation contours and affecting the extraction accuracy of phenotypic parameters such as root diameter and area. Moreover, most methods are trained and tested on only a single soil type, and their segmentation accuracy drops significantly in other soil backgrounds, indicating limited generalization ability. Whether introducing the wavelet transform into the segmentation network can alleviate the above problems and further improve root segmentation performance has not yet been validated.

In recent years, significant progress has been made in the study of unified spectral-spatial features in the field of hyperspectral image classification. SSUN achieves efficient feature extraction by jointly modeling spectral and spatial information [42]. SSUM introduces a state space model into hyperspectral image classification and achieves unified spectral-spatial feature modeling through spectral and spatial dual branches [43]. MSNAT proposes a multi-scale neighborhood attention Transformer that extracts multi-scale spatial features using local windows of different sizes [44]. DFTN revisits feature extraction from the frequency domain and captures high-frequency local detail features and low-frequency global smooth features through a dual-branch structure [45]. These works reveal the importance of synergistic modeling of frequency-domain information and spatial features.

Gómez-de-Mariscal et al. [46] introduced a user-friendly solution that enables the use of pre-trained deep-learning models for biomedical image analysis within ImageJ (https://imagej.net/ij/, accessed on 25 June 2026). Life science researchers often possess profound expertise in biological domains but may have limited familiarity with technologies such as deep learning. Therefore, designing a deep-learning-based tool accessible to non-specialists is crucial, as it can assist researchers in readily performing common image-processing tasks in life science studies, fostering interdisciplinary collaboration and the translation of research outcomes [47].

Specifically, current root phenotypic analysis still faces multiple challenges.

1. The segmentation accuracy under complex soil backgrounds is insufficient. The grayscale difference between fine roots and soil background is subtle, and there are interfering factors such as root occlusion and the similarity between roots and soil background. Existing methods such as U-Net and DeepLabV3+ use Max Pooling for downsampling, which inevitably loses high-frequency edge information of fine roots while reducing spatial dimensions, leading to missed detection of fine roots and blurred boundaries. Transformer-based methods such as TransUNet and SwinUNet have global modeling capabilities but are limited in capturing local detail features.
2. Root system morphology varies across different crops, and the quality and features of root images obtained through different acquisition methods also differ. Existing networks are often tailored to a single crop or a specific imaging technique, resulting in significantly decreased segmentation accuracy when applied to other datasets, with insufficient generalization ability and limited applicability.

To address the above problems, we propose an improved network model that replaces traditional Max Pooling with the Haar wavelet transform, thereby preserving multi-directional high-frequency detail features during downsampling and alleviating the issues of fine root missed detection and boundary blurring from the frequency domain perspective. At the same time, a multi-scale attention mechanism is introduced to suppress background noise interference during feature fusion and enhance the recognition capability for root structures of different scales. To improve the model’s generalization capability, we performed transfer learning on a mixed dataset, thereby boosting the model’s ability to segment root images from different imaging devices, various crop species, and diverse soil conditions. At the application level, the experiment designed a user-friendly interactive interface to lower the technical barrier, enabling more researchers without a computer science background to conveniently conduct root phenotypic analysis.

## 2. Materials and Methods

**Figure 1:**
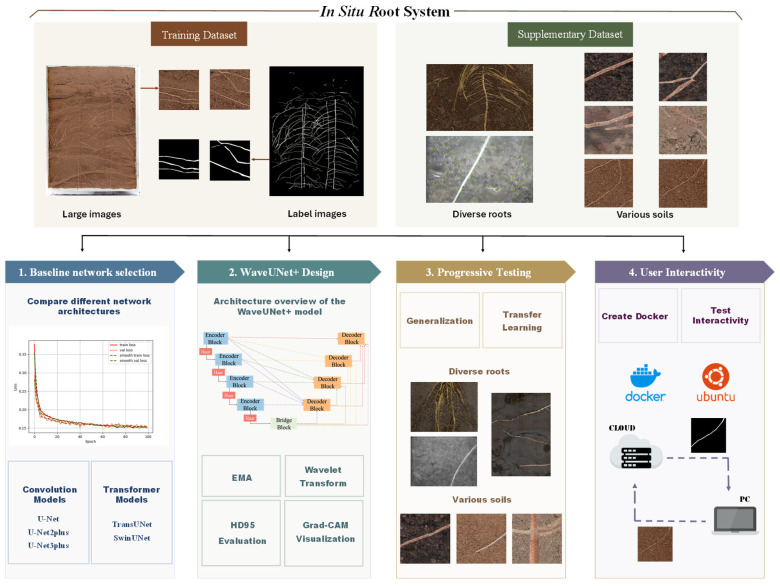
Processes related to the design of experiments.

Root image segmentation is susceptible to interference from complex backgrounds, and root structures exhibit multi-level characteristics, requiring models capable of capturing features at different scales. Furthermore, users often need specialized knowledge to configure environments and dependencies, which limits the practical application of models. Based on the above context, this experiment is divided into four main aspects (Figure 1). First, we identified a suitable base network for plant root segmentation. The experiment compared the segmentation performance of five networks on the same dataset. In addition to evaluation metric values and prediction results, factors such as model size, parameter count, and model inference time were also considered. Second, we designed the WaveUNet+ segmentation network and verified whether wavelet transform and attention mechanisms have a positive impact on plant root segmentation performance. Evaluation metrics of models incorporating wavelet transforms were compared, and Grad-CAM was used to visualize the segmentation performance of the models [48], providing an intuitive demonstration of the influence of wavelet transforms on the model’s focus. The multi-scale attention module enables focusing on image features at different scales, and full-scale skip connections allow the fusion of information from different hierarchical levels. The experiment employed HD95 to evaluate the impact of the attention mechanism on network segmentation performance [49]. An ablation study was conducted to determine the most suitable network combination for root segmentation. In addition to a subjective assessment of the final predicted images, an objective evaluation of model metrics was performed. Third, we tested the generalization capability of the WaveUNet+ segmentation model on diverse roots and various soils, and compared model performance before and after transfer learning. Finally, we packaged the model environment, dependencies, and network program into a fixed format through Docker deployment. This ultimately enables users to perform root phenotypic segmentation on plant root images from their own computers without needing to download complex software, install environments, or manage dependency libraries. The experiment also compared the prediction speed and total runtime for the same root images across a server, a local computer using Docker deployment, and an edge device.

### 2.1. Data Acquisition

#### 2.1.1. In Situ Root Image Collection

**Figure 2:**
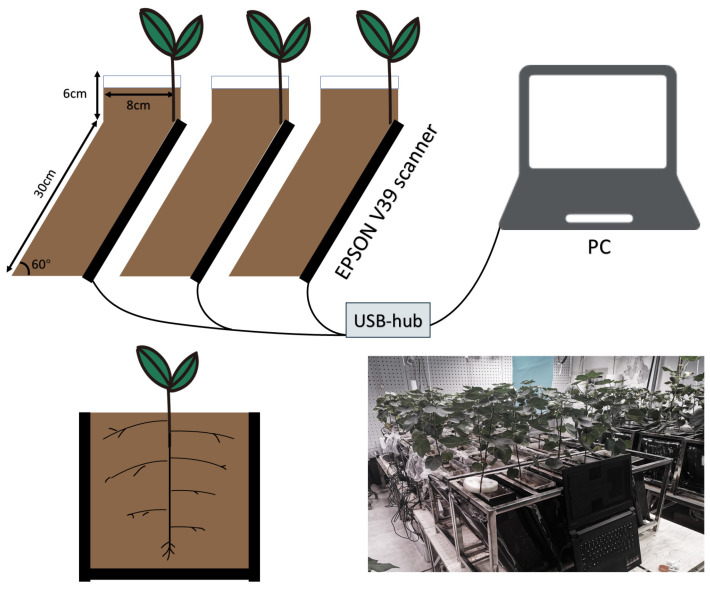
Schematic diagram of the RhizoPot working principle.

This experiment was conducted in 2023 at the experimental station of Hebei Agricultural University, Baoding City, Hebei Province (38.85° N, 115.30° E). Germinated cotton plants were cultivated in eight sets of RhizoPot devices, and images of cotton seedling root systems were continuously acquired over 110 days using an Epson scanner V39 (Epson Inc., Suwa-shi, Nagano, Japan) (Figure 2). RhizoPot (https://doi.org/10.3389/fpls.2022.1004904 accessed on 25 June 2026) is a plant cultivation container that uses soil as the growth medium to simulate field growth conditions. In the experiment, the RhizoPot was tilted at 60°. The scanner panel replaced one side of the acrylic plate and was positioned close to the growth medium to obtain high-standard images of the root system. The dimensions of the RhizoPot are 20 W × 8.5 D × 34 H cm^3^ (W, D, and H represent width, depth, and height, respectively) [15]. The cotton cultivar used was Guoxinmian 9. The growth medium consisted of a 3:1 mixture of 0–20 cm topsoil and sandy loam. After sowing, one seedling was retained per pot, and the RSWC (Relative Soil Water Content) was consistently maintained at 75 ± 5%. During the experimental period, each pot was weighed every other day to keep the RSWC within the set range. Images of cotton seedling roots were collected. The principles of the image acquisition device and the method for cultivating cotton seeds are described in previous articles from our research group. The acquired images had a resolution of 1200 dpi, a size of 10,200 × 14,039 pixels, and were saved in JPG format.

#### 2.1.2. Dataset Composition

One hundred images with fully developed roots, clearly visible structures, and no noise were selected as the dataset for annotation. Image annotation was performed by an experienced agronomy expert using the Lasso Tool in Adobe Photoshop CC (Adobe Inc., San Jose, CA, USA). All pixels identified as roots were marked in white and saved on a new layer, while the remaining pixels were marked in black. The annotation time for a single image was approximately five hours.

Due to the relative complexity of root systems in single large images, the large images were divided into smaller patches to capture fine details of the roots. For network training, the training, validation, and test sets were split in a ratio of 7:2:1. The resolution of each large image is 10,200 × 14,039 pixels, and each large image is cropped into approximately 137 sub-images, finally generating 13,694 pairs of training samples. The cropped sub-images exhibit diversity in spatial location, root density, soil texture, and other aspects. The divided label images and original images were retained as 24-bit images as part of the dataset. Through image processing methods, the dataset was segmented into images suitable for model training (1024 × 1024 pixels), yielding 13,694 original images and 13,694 label images. To better accommodate the input size of all models, the cropped images were resized from 1024 × 1024 pixels to 512 × 512 pixels, forming the original root dataset used for training the semantic segmentation network.

To validate the performance of the final model, the construction of the dataset primarily utilized public datasets as well as self-collected data (Table 1). For the use of public datasets, the experiment screened and integrated multiple high-quality open datasets related to plant root research [50]. Among them, to ensure a sufficient number of test samples for model generalization experiments, the PRMI (Plant Root Minirhizotron Imagery) dataset [51] and the HyperPRI dataset [52] were introduced. These datasets contain root images acquired using minirhizotron imaging devices and hyperspectral imaging equipment. A total of 2500 original images and 2500 label images were randomly selected to test the generalization ability of the model. In addition to testing the model on various plant roots obtained through different acquisition methods, we also captured root images under diverse soil environments. Specifically, we set up several different soil types, including black soil, sandy soil, and ordinary soil, to simulate a rich variety of soil conditions. Black soil was collected from the Songnen Plain in Northeast China (45° N, 120° E) and is a humus-rich soil with a dark brown color and containing a small amount of fine roots. The weight of black soil used in this experiment was 4.33 kg. Sandy soil was collected from the Yellow River (40° N) and consisted of river sand. The weight of sandy soil used in this experiment was 8.542 kg. Ordinary soil was collected from the experimental fields of Hebei Agricultural University in Baoding City, Hebei Province (39° N, 114° E). The weight of ordinary soil used in this experiment was 8.66 kg. Fifty images were selected from these as an additional test set.

### 2.2. Improved Strategies for In Situ Root Semantic Segmentation

#### 2.2.1. Base Network Selection

**Figure 3:**
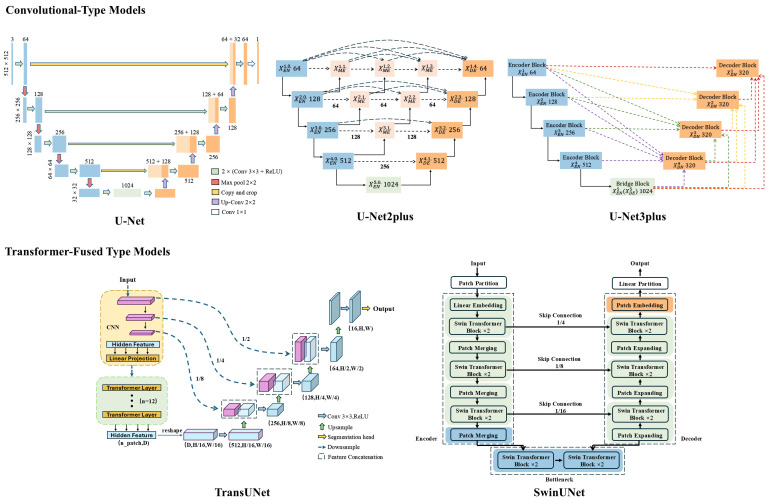
Structure diagram of base models.

To identify the most suitable base network for plant root segmentation, five networks were trained on the same dataset. Their evaluation metrics were compared, while model size and parameter count were also considered (Figure 3). The networks—U-Net2plus, U-Net3plus, TransUNet, and SwinUNet—all build upon the U-shaped architecture of U-Net. They incorporate improvements in feature fusion, global feature modeling, and Transformer-based architectural optimization, respectively, to better accommodate the requirements of segmentation tasks.

TransUNet introduces Transformer modules to enhance the ability to capture global context; however, its structure, which fuses CNN local features with Transformer global features, is relatively complex, leading to higher computational costs. SwinUNet is a U-shaped segmentation network based on Swin Transformer, capable of balancing global modeling, but it exhibits insufficient segmentation for fine roots and root-edge details. U-Net, as a commonly used semantic segmentation model, adopts an encoder–decoder architecture with same-scale skip connections, resulting in a relatively simple structure and efficient training. However, it also suffers from inadequate multi-scale feature fusion. U-Net2plus improves the feature fusion strategy of U-Net, enabling effective recognition of detailed features in root images. U-Net3plus enables each layer of the decoder to integrate features from all scales of the encoder, achieving more comprehensive multi-scale feature interaction, which further enhances the segmentation accuracy of root targets.

#### 2.2.2. Haar Wavelet Downsampling

The wavelet transform is a mathematical tool for signal processing and data analysis, serving as an extension and enhancement of the Fourier Transform. While the Fourier Transform can convert a signal from the time domain to the frequency domain, revealing its frequency components, it cannot simultaneously provide localized information of the signal in both the time and frequency domains. In contrast, the wavelet transform offers excellent localization properties in both the time and frequency domains, enabling the analysis of signal characteristics across different time scales and frequencies.

In convolutional neural network architectures such as U-Net and U-Net3plus, downsampling is typically performed using Max Pooling, which reduces the spatial dimensions of feature maps by selecting the maximum activation value within each pooling window. In in situ root images, the diameter of fine roots is usually less than 1 mm, occupying only 2 to 5 pixels in a 512 × 512 image. Their discriminative features are concentrated in the high-frequency components of the image. However, Max Pooling inevitably discards a large amount of high-frequency detail information because it only retains the maximum activation value within each local region. In in situ root images, the grayscale difference between lateral roots or root tips and the soil background is very subtle, and such information is mainly reflected in the high-frequency components. Once these high-frequency features are discarded during the downsampling stage by Max Pooling, the decoder cannot recover these critical details, leading to problems such as broken fine root segmentation and blurred boundaries.

**Figure 4:**
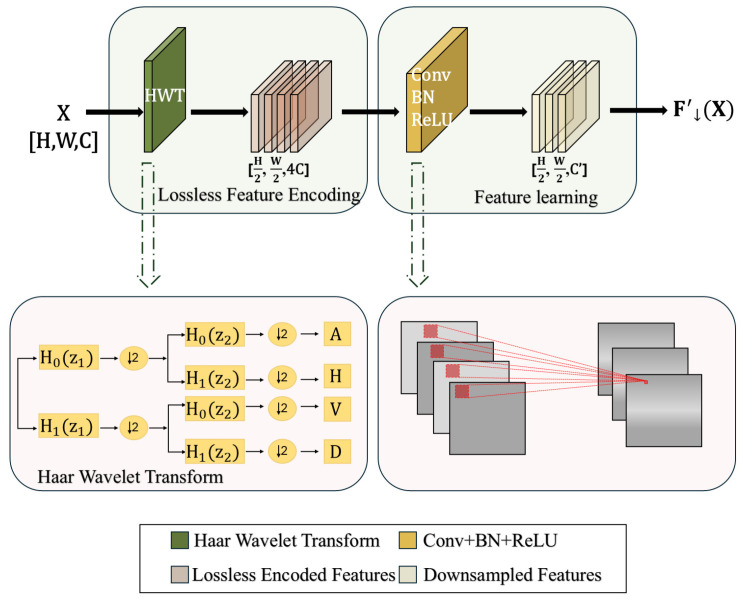
Schematic diagram of the Haar wavelet transform.

To address the above problems, this paper proposes using the Haar wavelet transform to replace Max Pooling for downsampling. The wavelet transform is based on wavelet functions, which are oscillatory functions with finite duration and rapid decay properties. Among them, the Haar wavelet is a commonly used wavelet function. In the image domain, the two-dimensional Haar wavelet transform decomposes an image into four sub-bands: LL (low-frequency–low-frequency), LH (low-frequency–high-frequency), HL (high-frequency–low-frequency), and HH (high-frequency–high-frequency). While Max Pooling retains only the maximum value in each local region, resulting in the loss of much background information, the Haar wavelet transform decomposes an image into low-frequency and high-frequency components, preserving more detailed information and thereby aiding the network in better learning image features (Figure 4).

The Haar scaling function $\phi \left(t\right)$ is defined on the interval [0, 1), and its formula is:

(1) $$\phi \left(t\right)=\left\{\begin{array}{c} 1,\;\;0\leq t<1\\ \;0,\;\;otherwise \end{array}\right.$$

The Haar wavelet function $\psi \left(t\right)$ is derived from the Haar scaling function, and its formula is:

(2) $$\psi \left(t\right)=\left\{\begin{array}{c} 1,\;\;\;\;\;\;0\leq t<\frac{1}{2}\\ -1,\;\;\;\;\frac{1}{2}\leq t<1\\ 0,\;\;\;\;\;otherwise \end{array}\right.$$

A one-level two-dimensional Haar wavelet transform is applied to the input image, yielding one low-frequency sub-band (LL) and three high-frequency sub-bands (LH, HL, HH). The low-frequency sub-band and high-frequency sub-bands are concatenated along the channel dimension, followed by a convolutional layer to adjust the channel count, ensuring compatibility with subsequent network layers. By replacing Max Pooling, the Haar wavelet transform achieves downsampling while fully preserving multi-scale features, thereby providing a richer informational foundation for feature fusion in the subsequent decoding stage.

#### 2.2.3. Multi-Scale Attention Module

In previous improved U-Net experiments, attempts were made to incorporate CBAM (Convolutional Block Attention Module) at the skip connections. It was observed that models enhanced with attention mechanisms could to some extent suppress features from irrelevant channels such as soil background and illumination noise, while strengthening geometric structures like root branches and root tips, as well as biological characteristics such as root color and texture [53,54]. When root and soil colors are similar, attention mechanisms can amplify the response of faint boundaries. However, the aforementioned attention mechanisms tend to focus more on high-contrast or large-scale structures such as the main root, while their ability to capture fine details like slender lateral roots or root hairs is relatively weak. In plant root segmentation tasks, accurate identification of these fine structures is critical for overall precision. Therefore, this experiment aims to identify an attention mechanism more suitable for plant root detection. In contrast, the EMA (Efficient Multi-scale Attention) module adopts a channel grouping parallel multi-scale strategy, dividing the channel dimension of the input features into several subgroups. Each subgroup extracts feature information at different scales using convolution kernels of different sizes, and then achieves adaptive fusion of multi-scale features through cross-dimensional interaction.

**Figure 5:**
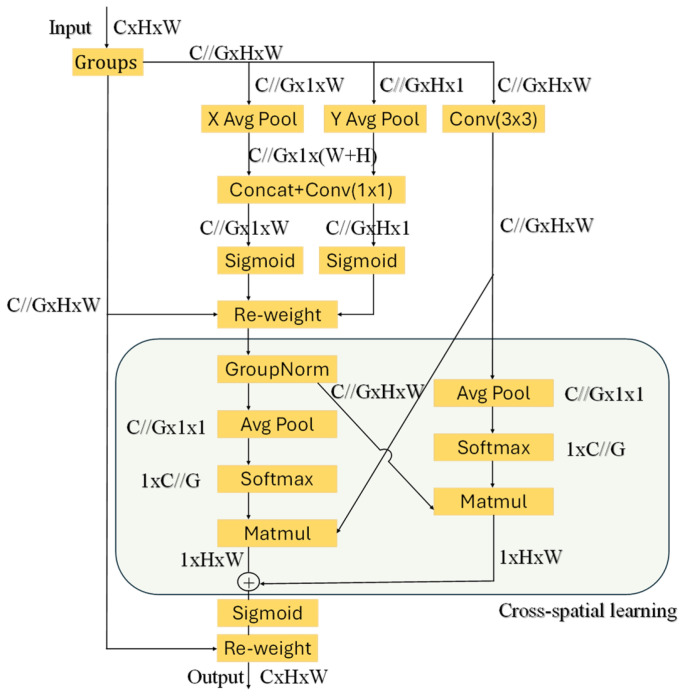
Schematic diagram of the EMA Module.

The EMA module is an attention mechanism designed for efficiently capturing multi-scale feature information. It enhances the model’s performance when handling information at different scales while reducing computational resource consumption [55]. Figure 5 illustrates the detailed structural diagram of the EMA module. The encoder part of the network architecture produces feature maps at various scales, and EMA can effectively fuse these multi-scale features, enabling the model to extract useful information across different hierarchical levels. Although the Haar wavelet transform enriches features, it introduces substantial redundant high-frequency noise, whereas EMA can suppress irrelevant high-frequency noise. Plant root images contain objects of various sizes, shapes, and textures. Experiments have shown that incorporating EMA at each fusion stage of the decoder helps the network model better adapt to these complex scenarios and achieve more precise segmentation of targets at different scales.

#### 2.2.4. WaveUNet+

In the original U-Net, skip connections only transfer feature maps from the encoder at the same scale to the corresponding layer in the decoder. In contrast, U-Net3plus adopts full-scale skip connections, which fuse feature maps from different scales of the encoder with the corresponding decoder layers. This approach enables the decoder to access multi-scale contextual information, thereby enhancing its ability to recognize targets of varying sizes. By implementing full-scale skip connections, U-Net3plus achieves more comprehensive feature interaction and more flexible contextual modeling. While retaining the simplicity of the original U-Net, it significantly improves segmentation performance for complex structures and multi-scale targets.

Since the original U-Net3plus also relies on Max Pooling for downsampling, which reduces feature map dimensions but discards high-frequency details, the model may introduce redundant information, potentially diminishing its discriminative capability for root targets. The incorporation of a multi-scale attention mechanism can compensate for this shortcoming. Haar wavelet downsampling supplements high-frequency details, reducing segmentation blur, while the multi-scale attention mechanism helps the network filter features, allowing the wavelet transform to provide valuable detailed information. The wavelet transform and the multi-scale attention mechanism complement each other, addressing both the detail-loss issue in U-Net3plus downsampling and the feature-redundancy problem in skip connections.

**Figure 6:**
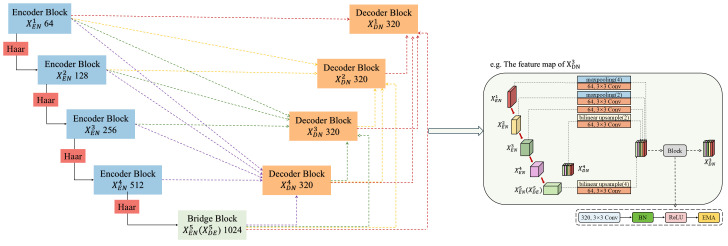
Architecture overview of the WaveUNet+ model.

Therefore, we propose a novel wavelet-enhanced full-scale segmentation network, termed WaveUNet+. Building upon the full-scale skip-connection framework of U-Net3plus, the model replaces Max Pooling with the Haar wavelet transform for downsampling to capture more detailed features. Additionally, the EMA attention mechanism is introduced at each fusion stage of the decoder to optimize cross-scale feature fusion. Figure 6 illustrates the network architecture of the WaveUNet+ model, specifically taking the third decoder layer as an example to demonstrate how the feature maps of $X_{DN}^{3}$ are constructed.

### 2.3. Model Performance Evaluation

#### 2.3.1. Evaluation Metrics

To objectively and accurately evaluate the performance of various networks, the experiment adopted multiple metrics, including mIoU, Recall, Precision, Dice, IoU, Loss, FLOPS, parameter count, and model size. Their calculation formulas are as follows.

(3) $$mIoU=\frac{1}{k+1}\sum_{i=0}^{k}\frac{P_{ii}}{\sum_{j=0}^{k}P_{ij}+\sum_{j=0}^{k}P_{ii}}\;$$

(4) $$Recall=\frac{TP}{TP+FN}=MPA$$

(5) $$Precision=\frac{TP}{TP+FP}$$

(6) $$Dice=\frac{TP}{TP+FP+FN}=F1$$

(7) $$IoU=\frac{2\times TP}{2\times TP+FP+FN}=\frac{Dice}{2-Dice}$$

(8) Loss=1−Dice

(9) $$FLOPS=\sum_{l=1}^{L}K_{l}^{2}\times C_{in,l}\times C_{out,l}\times H_{out,l}\times W_{out,l}$$

In Equation (3), the subscript i represents the ground truth, and j represents the prediction. In Equations (4) and (5), TP denotes true positives, i.e., the number of positive samples that are correctly predicted as positive—pixels predicted as roots that are actually roots. FN denotes false negatives—pixels predicted as background that are actually roots. FP denotes false positives—pixels predicted as roots that are actually background. TN denotes true negatives—pixels predicted as background that are actually background. This paper comprehensively analyzes seven metrics. The models are evaluated using a test set that was not used for training. The relevant parameter values are obtained by executing Python (Python 3.8) scripts for computation, and data statistics are performed using Excel.

#### 2.3.2. Grad CAM Model Visualization

Grad-CAM (Gradient-weighted Class Activation Mapping) is a technique for visualizing deep-learning models. By computing a weighted sum of the gradients and feature maps from the last convolutional layer, it generates a heatmap that visually highlights the regions the model focuses on when making decisions, thereby providing an intuitive interpretation of the model’s decision-making process. Its core formula is:

(10) $$\alpha_{k}^{c}=\frac{1}{z}\sum_{i}\sum_{j}\frac{\partial_{y^{c}}}{\partial_{A_{ij}^{k}}}$$

(11) $$L_{GradCAM}^{c}=ReLU(\sum_{k}\alpha_{k}^{c}A^{k})$$

$y^{c}$ represents the model’s output score for the root target, $A_{ij}^{k}$ denotes the pixel value of feature map $A^{k}$ at position (i, j), and Z indicates the spatial dimensions of the feature map. The weights are summed with the feature maps through weighted aggregation, and a ReLU activation is applied to focus on regions that positively influence the target.

In this experiment, Grad CAM was employed to test two models with downsampling performed by Max Pooling and Haar wavelet transform, respectively. Whether the attention regions of the model incorporating the wavelet transform shift significantly can be visually verified through heatmaps. The epoch with the optimal metric values during training was selected for in-depth visualization analysis, generating heatmaps for the feature maps corresponding to each up-sampling and down-sampling layer in the network.

To validate the advantages of the Haar wavelet transform, the experiment also compared the evaluation metrics of models incorporating different wavelet transforms. The tested configurations included the baseline network model (model1), a network with downsampling using the Haar wavelet transform (model2), a network with downsampling using the DB2 (Daubechies 2) wavelet transform (model3), and a network with downsampling via the Haar wavelet transform and the inverse wavelet transform incorporated into upsampling (model4).

The comparison between model1 and model2 tests whether incorporating the Haar wavelet transform can further improve the network’s root segmentation accuracy. The comparison between model2 and model3 aims to evaluate which wavelet yields better segmentation performance for roots. Although both DB2 and Haar wavelets belong to the family of orthogonal wavelets in wavelet analysis, they differ fundamentally in construction, mathematical properties, and time–frequency characteristics. Heatmaps can visually illustrate the respective impacts of the two wavelets on the network. Comparing model2 and model4 demonstrates whether the inverse wavelet transform also exerts a positive influence on the model’s segmentation performance.

#### 2.3.3. HD95 Evaluation Metric

The EMA attention mechanism enhances root-boundary prediction accuracy by dynamically filtering effective information across scales while suppressing background redundancy and noise interference. To comprehensively evaluate the improved model, this paper adopts HD95 (95th Percentile Hausdorff Distance) as a boundary-assessment metric before and after the introduction of the attention mechanism. HD95 quantifies the 95th-percentile shortest distance between the predicted segmentation mask and the GT (ground truth) boundary point set. Compared with the original HD (Hausdorff Distance), which is sensitive to outliers, HD95 excludes the extreme 5% of deviations, yielding more stable and reliable evaluation results. Its core formula is:

(12) $$HD95\left(A,B\right)=95th\;percentile(\left\{d\left(a,B\right)|a\in A\right\}\cup \left\{d\left(b,A\right)|b\in B\right\})$$

Among them, A is the boundary point set of the root segmentation mask predicted by the model, and B is the boundary point set of the manually annotated root image serving as the GT. $d\left(a,B\right)$ represents the shortest distance from a predicted boundary point a to the GT boundary point set B, and $d\left(b,A\right)$ denotes the shortest distance from a GT boundary point b to the predicted boundary point set A. The 95th percentile is then taken over all these bidirectional distances, which filters out the extreme 5% of outliers.

### 2.4. Model Generalization Evaluation

#### 2.4.1. Generalization Capability Test

Model generalization capability refers to the model’s ability to adapt to new data. Ideal generalization requires the model to extract general patterns from the training data rather than merely memorizing specific sample characteristics. Traditional deep-learning models are susceptible to data bias. To validate the generalization capability of our proposed WaveUNet+ model, we selected root images acquired via scanner devices and public datasets as the test set. The test images are specifically divided into two groups: diverse roots and various soils.

Diverse roots include complete cotton root systems (taproot systems), complete wheat (*Triticum aestivum* L.) root systems (fibrous root systems), peanut (*Arachis hypogaea* L.) root systems acquired via hyperspectral imaging, and root images of various plants, such as peanut, papaya (*Carica papaya* L.), and cotton, obtained through minirhizotron systems. Since cotton root systems were used as the training dataset, wheat—a typical fibrous-rooted plant—was selected to further validate the model’s adaptability to different root morphologies and to test whether root systems of other species can also be accurately recognized. The hyperspectral peanut root dataset highlights the spectral characteristics of plant roots but presents issues such as uneven illumination and noise interference. Similarly, the minirhizotron plant root dataset contains root systems of different crops, with backgrounds that may include soil particles and moisture reflections.

Furthermore, to examine whether our model possesses robust segmentation capability in complex scenarios such as plant root phenotypic analysis, various soils specifically include plant root images grown in black soil, sandy soil, and ordinary soil environments. Although soil characteristics such as color and moisture may be unrelated to root phenotypes, the model could mistakenly treat them as features.

#### 2.4.2. Transfer Learning Optimization Strategy

To further enhance the generalization capability of the improved model, the experiment introduced a transfer learning strategy for optimization. First, the model before transfer learning was used to perform segmentation predictions on root images from the diverse root and various soil categories, obtaining reference results for comparison. Subsequently, transfer learning was applied to the WaveUNet+ model. The pretrained backbone was frozen to prevent drastic changes on the new task, thereby preserving the general features learned from the original dataset, such as the recognition of root edges and textures. Since training for an excessive number of epochs can lead to overfitting, resulting in poor final predictions and reduced metric values such as mIoU and Precision, this experiment utilized transfer learning to further train the best-performing weights from the previous training stage. To balance model performance and overfitting risk, a dynamic training mechanism was adopted, setting the maximum number of training epochs to 10. During training, the segmentation performance on the validation set was monitored in real time, and the weight parameters with the best overall performance in each epoch were dynamically saved. The optimal weights were then used as the final parameters of the model.

The purpose of transfer learning is to verify whether the model can effectively suppress interference from irrelevant features and maintain stable segmentation performance when noise or distracting variables are introduced in the target domain. To comprehensively evaluate the effect of transfer learning, the WaveUNet+ model before transfer learning was first used to predict plant root images from the diverse root and various soil categories, serving as a baseline for comparison. Subsequently, the same set of images was predicted by the model after transfer learning, enabling a direct visual comparison of their segmentation performance in terms of root-boundary completeness. In addition to the subjective assessment of predicted images, the experiment employed a mixed dataset to compute evaluation metrics such as mIoU, Precision, and Dice before and after transfer learning, thereby providing an objective, quantitative assessment of model performance.

### 2.5. Practical Deployment

To enhance user-friendly interaction in the proposed method, we further tested the practical deployment feasibility of the model. Through containerization deployment and interface design, the complex deep-learning model is transformed into a ready-to-use tool that requires no environment configuration. This approach promotes interdisciplinary applications in root phenotypic research, lowers the technical barrier to usage, and enables researchers outside the deep-learning field to conveniently perform root image recognition and phenotypic analysis.

By packaging the environment dependencies through Docker, the complex environment, dependency libraries, and network programs are bundled into a fixed image. This ensures localized processing of user data for privacy and security, while lowering the technical barrier to usage, thereby facilitating adoption by agronomy-related researchers. Leveraging Alibaba Cloud Elastic Compute Service and Container Service enables cloud-hosted Docker container deployment, guaranteeing cross-platform compatibility and environment consistency. Ubuntu serves as the server-side operating system, providing a stable runtime environment for the instance, supporting GPU drivers, and enabling seamless integration into Windows environments via WSL2 (Windows Subsystem for Linux 2).

The experiment compared the prediction time and speed of the WaveUNet+ model deployed on a server, on a Raspberry Pi, and via Docker on a local computer. This comparison was conducted to validate the model’s applicability and practical deployment feasibility. The advantage of using Docker deployment lies in its environment isolation and cross-platform consistency, which addresses issues arising from configuration differences across devices that could otherwise cause abnormal model operation. Comparing the speed difference between Docker deployment on a local computer and on a server allowed us to test the impact of Docker encapsulation on prediction efficiency. Meanwhile, deployment testing on the Raspberry Pi further verified the practical viability of the model, providing hardware support for in situ root segmentation in field conditions.

### 2.6. Experimental Design

This experiment was conducted using both Windows and Linux systems. The GPU models were a 3080Ti-12G and a 3090Ti-24G, with video memory of 12 GB and 24 GB, respectively. The CPU was an AMD Ryzen 7 3700X 8-Core Processor (Advanced Micro Devices, Inc., Santa Clara, CA, USA). The training image size was 512 × 512 pixels. The original images were 24-bit JPG files, and the label images were 24-bit PNG files, both with a horizontal and vertical resolution of 96 dpi. The dataset consisted of 13,694 original images and 13,694 label images, divided into training, validation, and test sets. For transfer learning, a mixed dataset was used that included root images of different plants acquired via minirhizotron, hyperspectral imaging, and scanner methods. This mixed dataset contained 16,194 original images and 16,194 label images, with the training, validation, and test sets again split in a 7:2:1 ratio. The hyperparameter configurations for both training phases are detailed in Table 2.

To identify the most suitable base network for root segmentation, this paper compared the segmentation performance metrics of various networks. The optimal model should not only excel in segmentation effectiveness but also consider model size and parameter count. Using the skip connections of the original U-Net as a baseline, we verified whether full-scale skip connections could perform better in plant root segmentation tasks. The experiment tested the overall impact of incorporating different wavelet transforms on the network models. Heatmaps generated via Grad-CAM were used to visualize the segmentation performance of the models. The EMA module enhances the effectiveness of multi-scale feature fusion, and its contribution to the network was validated using the HD95 boundary evaluation metric. An ablation study was also conducted on the proposed model, comprehensively analyzing the overall impact of each module on the network based on both segmentation results and evaluation metrics.

Next, this paper validates the generalization capability of the proposed model on both diverse roots and various soils. The experiment compared the segmentation results before and after transfer learning and computed the evaluation metrics on a mixed dataset. To further enhance interactive usability, the experiment containerized the user-interface environment via Docker deployment and also tested on-board deployment on the edge device, Raspberry Pi.

## 3. Results and Discussion

### 3.1. Performance Validation

#### 3.1.1. Base Network Comparison

Five segmentation networks—U-Net, U-Net2plus, U-Net3plus, TransUNet, and SwinUNet—were compared as base networks. Each model was trained on the same dataset, and its segmentation performance and lightweight characteristics were comprehensively evaluated using the metrics mIoU, Precision, Recall, Dice, IoU, Loss, Parameters, and Size. The specific numerical results are presented in Table 3.

Root image segmentation requires the model to accurately capture fine details and edges. The multi-scale fusion in U-Net2plus and U-Net3plus compensates for the limitations of the original U-Net, and both models outperform the original U-Net in metrics such as mIoU and Precision. U-Net3plus employs 1 × 1 convolutions to reduce the channel dimensions of features at each scale before fusion, whereas U-Net2plus uses nested connections without targeted channel compression. As a result, U-Net2plus exhibits higher values in both Parameters and Size compared to U-Net3plus.

The global attention mechanism relied upon by Transformer is susceptible to interference from complex soil backgrounds, making it difficult to accurately capture local edges and branch details of root systems. TransUNet and SwinUNet performed poorly in plant root segmentation. The mIoU of TransUNet was 84.80%, which is lower than that of the U-Net model, while its parameter count and model size are substantially higher than those of other models. SwinUNet achieved an mIoU of only 67.09%, and its Recall was significantly lower compared to the other four models. The Loss value of U-Net3plus was 7.78%, lower than that of the other four models, indicating more stable convergence during training with less susceptibility to overfitting or gradient fluctuations. In contrast, the Loss value of SwinUNet was notably higher, further demonstrating its limited suitability for root segmentation tasks.

The results indicate that among the five base networks, U-Net3plus achieves the best overall performance. Its mIoU, Precision, Dice, and other metrics are higher than those of the other network models, with an mIoU of 86.39% and a Dice score of 92.22%, which are significantly higher than those of the other models. Moreover, it has the smallest parameter count and model size. Thus, for root image segmentation, U-Net3plus demonstrates the best performance, achieving an optimal balance between the highest accuracy and the smallest model footprint, thereby meeting the practical requirements for subsequent transfer learning and edge-device deployment.

Table 4 presents the supplementary test results of four additional segmentation frameworks. Although the Mamba U-Net network model [56] has a fast training speed, it struggles to learn plant root features. After 100 training epochs, it still cannot separate plant roots from the background. The global modeling capability of Mamba may introduce random noise in the root segmentation task, while its local feature extraction ability is insufficient to distinguish roots from soil. We also tested the UKAN segmentation model [57]. The experimental results show that UKAN performs poorly in plant root segmentation and cannot effectively capture the fine details of roots. Although UKAN achieves a low loss, its best IoU is only 72.71%, which is 9.52 percentage points lower than U-Net and 12.86 percentage points lower than U-Net3plus. DeepLabV3+, as a representative method based on dilated convolutions and the ASPP module, achieves an IoU of 81.59%, which is 3.98 percentage points lower than U-Net3plus. Although DeepLabV3+ has a strong multi-scale feature extraction capability, its strategy of expanding the receptive field with dilated convolutions is less effective for fine root segmentation than the full-scale feature fusion mechanism of U-Net3plus. CBAM-UNet achieves an IoU of 84.49%, outperforming DeepLabV3+ but still 1.08 percentage points lower than U-Net3plus. U-Net3plus, with its full-scale skip connection feature fusion mechanism and superior overall performance, performs best among all candidate baseline networks. Therefore, U-Net3plus is selected as the baseline network for this work.

#### 3.1.2. Validation of Haar Wavelet Downsampling

The role of Haar wavelet downsampling was evaluated from two perspectives. First, the experiment conducted a visual analysis using Grad-CAM heatmaps to directly compare the feature-focus regions of the models. Second, the experiment objectively compared the evaluation metrics of models incorporating different wavelet transforms.

**Figure 7:**
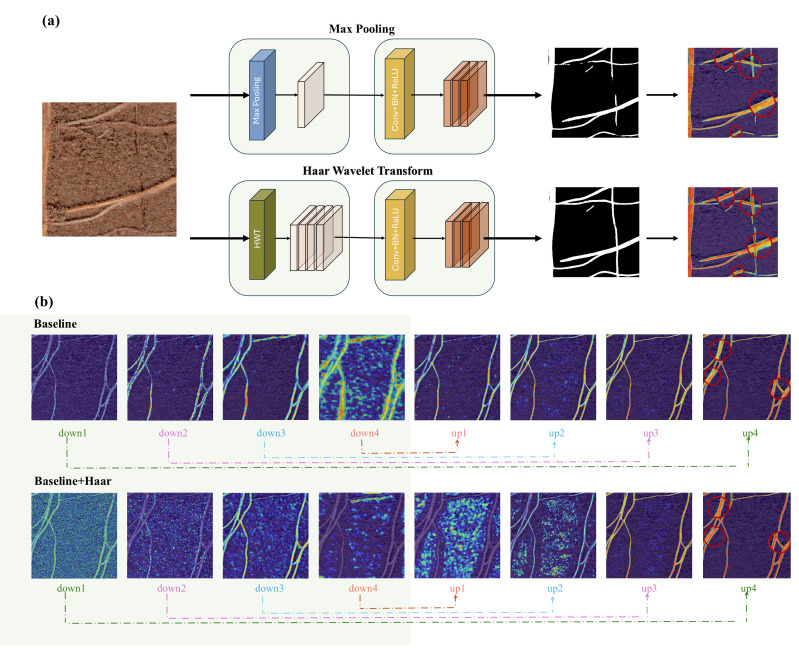
(**a**) Comparison of downsampling methods. (**b**) Comparison of heatmap visualization. The red circle indicates the magnified area, showing the detailed differences between the models.

Figure 7a illustrates the structural differences between the Max Pooling and Haar wavelet transform networks, comparing their segmentation results and corresponding visualized heatmaps. The Max Pooling module compresses feature dimensions solely through pooling operations, outputting a single-channel feature representation. In contrast, the Haar wavelet transform decomposes the input into multi-channel features, providing richer initial feature representations. When the network uses Max Pooling for root segmentation, the pooling operation loses the high-frequency gradient information at the edges of fine roots. As a result, the model cannot effectively distinguish fine roots from the soil background and misclassifies fine root pixels as background, leading to root breakage in the segmentation results. In contrast, the Haar wavelet transform preserves the LH, HL, and HH high-frequency sub-bands, effectively retaining edge details in the horizontal, vertical, and diagonal directions. To visually highlight the impact of the wavelet transform on the network, a magnified view of the heatmap is included, with red circles marking detailed regions. When using Max Pooling for root segmentation, the model is more easily influenced by the soil background, mistakenly identifying root regions as background and resulting in broken roots in the segmentation output. Warmer colors in the heatmap indicate higher model attention to the corresponding regions. Compared to Max Pooling, the Haar wavelet transform shows greater overall attention to root targets and yields better segmentation of fine root details.

Figure 7b compares the performance visualization heatmaps of the Baseline and Baseline + Haar models at each layer (down1–down4, up1–up4). The comparison of the two models demonstrates the positive impact of the Haar wavelet transform on the overall focus of the model. The experiment revealed a clear difference in their focus regions. At down1, the incorporation of the wavelet transform assists the model in attending to the image globally. Compared to the Baseline model, the heatmaps of the model with the Haar wavelet transform shift more attention from the second down-sampling layer to the first up-sampling layer toward regions distinct from the root area, introducing background detail features. This helps the model balance information between roots and background when handling complex scenes. At up1 and up2, the Baseline model, using Max Pooling, focuses only on the roots and neglects the boundaries between roots and background, whereas the Baseline + Haar model is able to attend to both the background boundaries and the root structures themselves. In the subsequent three up-sampling layers, the model with the wavelet transform progressively strengthens its focus on the roots, ultimately shifting its attention from the background boundaries toward the plant roots. The Baseline model only reinforces its focus on the roots while overlooking the supporting role of background information for root segmentation. A magnified view of the up4 heatmap is provided, with detailed areas marked. The Haar wavelet emphasizes the extraction of edges, textures, and other fine details. When the Haar wavelet transform is incorporated, the model attends not only to the characteristic features of plant roots but also captures structural information from the background during the feature extraction stage, ultimately better capturing the root targets and achieving the best segmentation performance at up4.

After confirming the overall impact of the wavelet transform on the network, an attention mechanism was introduced into the wavelet-enhanced network. Heatmaps for each up-sampling and down-sampling layer were analyzed using weights saved from epoch 10 to epoch 100 at intervals of 10 epochs, and were also compared with the heatmaps generated from the weights of the best-performing epoch. This allowed us to visually examine how the training epoch influences the model’s focus regions. It was observed that as the epoch number increased, the focus regions became progressively more stable, and the energy concentrated on the plant roots in the final prediction output gradually increased (see Supplementary Material).

**Figure 8:**
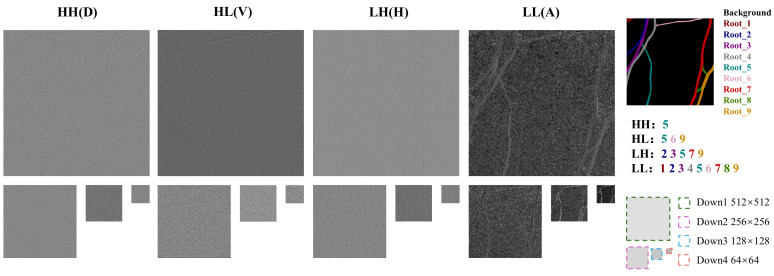
Wavelet frequency-decomposition images.

Figure 8 compares the wavelet sub-band output images of each down-sampling layer (down1–down4) in the Baseline + Haar model. The image resolution gradually decreases with deeper down-sampling layers. LL is the low-frequency sub-band, which primarily preserves the main outline of the image; HH, HL, and LH are high-frequency sub-bands that retain edge and detailed features. The differences in gray-level distribution among HH, HL, and LH in the figure reflect the varying ability of different layers and sub-bands to capture details. Furthermore, the root system in the image is divided into nine independent branches, labeled Root1 to Root9 and displayed in nine distinct colors. This illustrates the detailed focus of each sub-band on individual branches, visually demonstrating the ability of each wavelet sub-band to extract local root information. This validates that the multi-subband decomposition of the Haar wavelet transform can comprehensively capture root morphological information from different directions and frequencies, achieving the multi-scale frequency analysis capability that Max Pooling cannot provide. Currently, the wavelet sub-bands are reconstructed by simple concatenation. In later stages, the proportional relationships among the sub-bands could be adjusted, and weighting coefficients for different sub-bands may be explored to achieve an optimal reconstruction, thereby further highlighting the advantages of wavelet transform in processing high-frequency information.

Table 5 compares four models, including the U-Net3Plus baseline model (model1), the network with downsampling using the Haar wavelet transform (model2), the network with downsampling using the DB2 wavelet transform (model3), and the network with downsampling via the Haar wavelet transform and the inverse wavelet transform incorporated into upsampling (model4).

The comparison showed that the model incorporating Haar wavelet transform for downsampling yielded better overall metrics, achieving the highest mIoU among all wavelet-transform variants at 86.42%. The experiment also tested replacing the Haar wavelet transform with the DB2 wavelet transform for downsampling. The results indicated that the network using the DB2 wavelet performed worse overall than the Haar-based model, with Precision lower by 0.74%, Recall lower by 0.64%, and Dice reduced by 0.68%. Model3 showed the lowest Precision value among the compared models and was more susceptible to background interference, frequently misclassifying background as roots. Additionally, the experiment attempted to incorporate the inverse wavelet transform for upsampling after applying the Haar wavelet transform in downsampling. However, compared with the model without the inverse transform, Precision decreased by 0.62% and Recall dropped by 0.39%, while both model size and parameter count increased. Model4, which included the inverse wavelet transform, exhibited weaker detail-handling capability compared to Model2 and showed deficiencies in segmentation accuracy and the ability to process complex structures.

#### 3.1.3. Multi-Scale Image Segmentation Performance Evaluation

Table 6 compares the impact of two different attention mechanisms, CBAM and EMA, on network performance. Using U-Net3plus with Haar wavelet as the baseline comparison model, the CBAM and EMA modules were respectively introduced at the feature fusion stage of the network. For each model, we calculated mIoU, Precision, Recall, Dice, IoU, and Loss, and also evaluated HD95 on the same 105 root images.

The model with EMA achieves the highest mIoU, Dice, and IoU among the three groups, as well as the lowest Loss. EMA significantly outperforms CBAM in overall metrics. The model with EMA attains a Recall of 92.33%, which is 0.43% higher than that of CBAM and 1.46% higher than that of U-Net3plus + Haar. Recall effectively measures the detection rate of root pixels; its improvement indicates that more fine lateral roots are correctly identified, demonstrating that the multi-scale parallel perception mechanism of EMA enhances the representation of fine root structures. The model with CBAM shows a Precision decrease of 0.99% compared to U-Net3plus + Haar, suggesting that while CBAM enhances fine root detection, it also misclassifies some soil background as roots. In contrast, although the Precision of the model with EMA is also lower than that of U-Net3plus + Haar, it achieves the greatest improvement in Recall while maintaining relatively high Precision, striking a good balance between detection rate and precision.

CBAM has insufficient adaptability to the multi-scale structure of plant roots. In contrast, EMA extracts multi-scale features by grouping channels in parallel, allowing it to simultaneously focus on root structures at different scales. When combined with the multi-subband output of the Haar wavelet transform, EMA can selectively filter effective information from each of the LL, LH, HL, and HH sub-bands, achieving refined utilization of frequency-domain features.

The experiment selected 105 cotton root images with a resolution of 512 × 512 pixels as the test images. HD95 was adopted as the evaluation metric for boundary accuracy of the segmentation results. A lower HD95 value indicates higher overlap between the predicted root contour and the ground truth annotation, i.e., higher segmentation accuracy. The U-Net3plus + Haar model achieved an HD95 of 5.15, while the model with the EMA module reduced the HD95 to 4.48. These results demonstrate that, on the basis of using wavelet transform for downsampling, further introducing the EMA module at each fusion stage of the decoder not only improves model accuracy metrics such as mIoU, Recall, and Dice, but also makes the predicted root contours more closely match the true morphology.

**Figure 9:**
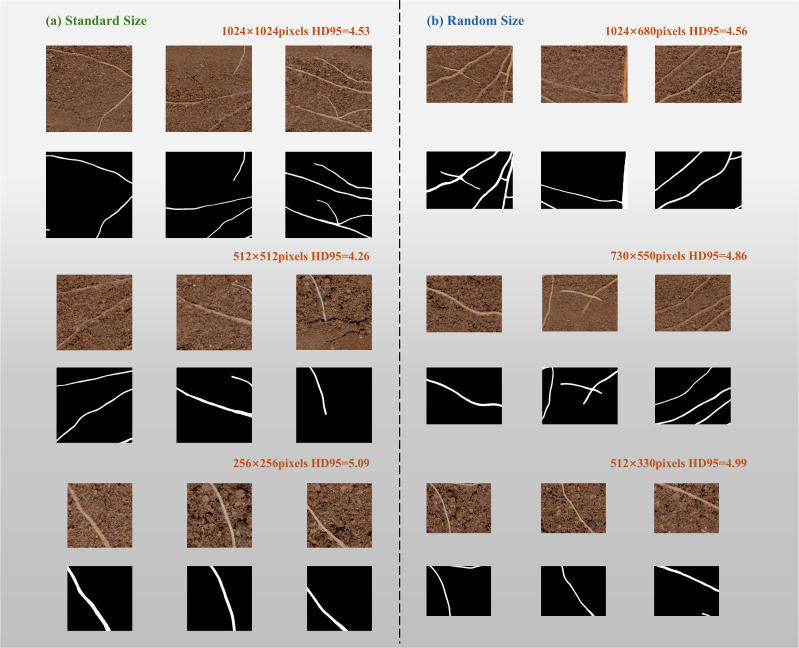
Multi-size image segmentation results.

Furthermore, the experiment tested the segmentation capability of the final improved model on images of different sizes. Images with regularly sized dimensions Figure 9a and randomly sized dimensions Figure 9b were selected, with three images per size category used for root-target segmentation. The mean HD95 value was calculated for each group. As shown in Figure 9a, the network performed best on the standard 512 × 512 pixel size that matches the training dimensions, achieving an HD95 value of 4.26. The model exhibits a higher degree of feature fitting to the training scale compared to images of other sizes. Even when handling images with other regular sizes or random sizes, the model’s HD95 values remained within a favorable range. This further demonstrates that the dynamic optimization of multi-scale features by the EMA module effectively enhances the model’s adaptability to root images of varying dimensions.

When U-Net3plus fuses multi-scale features, it does not distinguish between useful features and redundant ones, which can easily lead to background noise or irrelevant features being incorporated into the fusion process. In contrast, EMA dynamically filters the effective information across scales.

### 3.2. Ablation Study

#### 3.2.1. Segmentation Effect Comparison

Our WaveUNet+ model builds upon the full-scale skip-connection framework of U-Net3plus, integrates wavelet transform to capture fine-grained details, and incorporates the EMA module to optimize cross-scale feature fusion. The Haar wavelet transform is a signal-processing technique that can decompose a signal into sub-bands of different frequencies. Without substantially increasing computational cost, it better preserves detailed image information, thereby enhancing the model’s segmentation performance. The full-scale skip-connection design in U-Net3plus enables cross-level feature fusion, but the conventional concatenation approach lacks the ability to distinguish the importance of different features. In contrast, the EMA attention mechanism dynamically assigns weights, effectively suppressing background noise interference when features are transmitted from the encoder to the decoder.

Since the model incorporates multiple different modules, an ablation study was conducted to investigate the impact of each module on segmentation performance and to validate the effectiveness of the combined strategy of wavelet transform and attention mechanisms. The experiment compared four different network configurations to observe specific changes in model performance through the ablation study.

**Figure 10:**
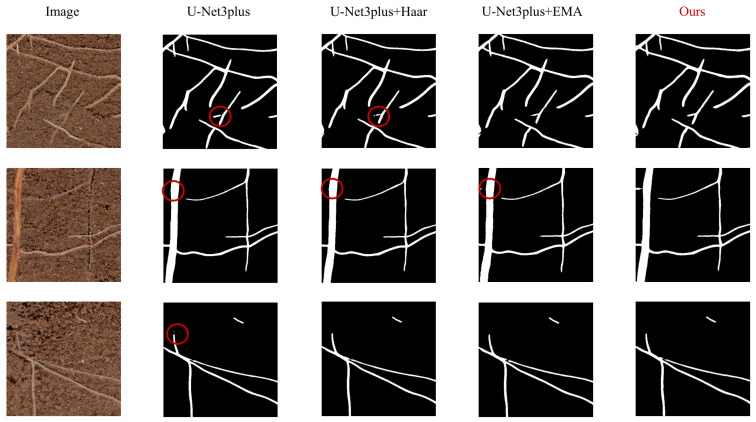
Comparison of original images and segmentation results from different models. The red circles indicate the detailed differences among different segmentation models.

Figure 10 marks with red circles the regions where each model exhibits poor segmentation performance or is prone to errors. By comparing the segmentation results of different models, it can be observed that although U-Net3plus extracts the overall spatial structure of roots reasonably well, it still suffers from many detailed segmentation problems. Due to the small width and low contrast of fine roots, the traditional downsampling process tends to lose high-frequency detail information, leading to obvious fine root breakage. For low-contrast fine roots with soil background textures that are similar, the model struggles to distinguish the boundaries, causing some fine root regions to be misclassified as background and some non-root background regions to be incorrectly identified as roots, resulting in both missed detection of fine roots and false detection of background. Bright particles, cracks, or texture edges in the soil resemble root morphology, and U-Net3plus misidentifies these non-root regions as roots in the absence of effective attention constraints. At root intersections, the boundary relationships between different roots are complex, and the model may make errors in determining the intersecting regions and branch directions, or suffer from missing local structures. Moreover, due to soil occlusion, root continuity is disrupted, and the model finds it difficult to accurately recover the connections in occluded regions.

The high-frequency detail features preserved by the Haar wavelet transform effectively alleviate the information loss caused by downsampling. Compared to the U-Net3plus model, the occurrence of broken roots and missing segmentation is reduced. However, this model does not address the issue of background interference during the feature fusion stage, and some loss of detail along root edges remains. The U-Net3plus + EMA model achieves better segmentation of the main root structure. The introduction of EMA can effectively reduce the false detections caused by background noise, but it cannot compensate for the lost detail features, indicating that improving a single module has its limitations.

Our model combines the strengths of both modules, resulting in superior handling of fine root details and more complete segmentation of delicate root structures compared to other models. It also demonstrates superior performance in fine-root continuity, recognition of root intersections, and background noise suppression, yielding segmentation results that are more complete, more continuous, and closer to the true root structure. However, when roots are occluded by soil, the model can still produce broken segments. Future research will consider reconstructing root systems to represent their truest form within the soil.

#### 3.2.2. Evaluation Metric Comparison

Evaluation metrics enable an objective analysis of the segmentation performance of each model. Table 7 provides a numerical comparison of the segmentation effectiveness of the models using common metrics such as mIoU, Precision, Recall, and Dice. Using U-Net3plus as the baseline model, the dataset was randomly divided five times. The relevant parameter values were computed by running a Python program, and data statistics were completed using Excel. The results are presented as mean ± standard deviation.

The experiment compared four different model configurations. From the numerical results, although the U-Net3plus model performed best in terms of parameter count and model size, its overall metric values were relatively inferior. While improving Recall may affect Precision to some extent, it is essential to balance the trade-off between them. The Dice score effectively balances Recall and Precision, providing a comprehensive evaluation. Our improved model enhances the overall evaluation metrics without substantially reducing Precision. It achieves the highest Dice among the compared models, while also maintaining relatively low parameter count and model size. Specifically, the WaveUNet+ model attains a mean mIoU of 87.08% over five random data splits, which is 1.52 percentage points higher than the original U-Net and 0.39 percentage points higher than the original U-Net3plus. Its mean Recall is 92.34%, representing an increase of 2.93 percentage points over the original U-Net and 1.61 percentage points over the original U-Net3plus. WaveUNet+ has FLOPs of 814.53 G, which is only 14.27 G higher than the baseline, and an inference speed of 5.00 it/s, which is 0.60 it/s lower than the baseline. Considering both accuracy and efficiency, WaveUNet+ achieves improvements in mIoU, Recall, Loss, and other metrics while maintaining relatively low computational cost, demonstrating a favorable accuracy–efficiency trade-off.

When the downsampling stage is replaced by the Haar wavelet transform, the network can simultaneously preserve spatial location information and multi-scale frequency-domain features while extracting root characteristics. This enhances the recognition of fine roots against soil backgrounds, reduces missed detections caused by feature loss, and effectively improves the values of mIoU and Recall. The introduction of the EMA module suppresses interfering information from complex backgrounds, thereby enhancing the classification accuracy of the model and directly contributing to the optimization of the mIoU metric

By integrating both modules, the model leverages the feature extraction capability of the Haar wavelet transform to strengthen the acquisition of information from fine root regions, while the EMA module filters the effective information, reducing interference from background noise on segmentation. Our model not only achieves the best performance in mIoU, Recall, Dice, and IoU but also attains the lowest Loss value, indicating that the WaveUNet+ model possesses superior segmentation ability compared to other combinations.

**Figure 11:**
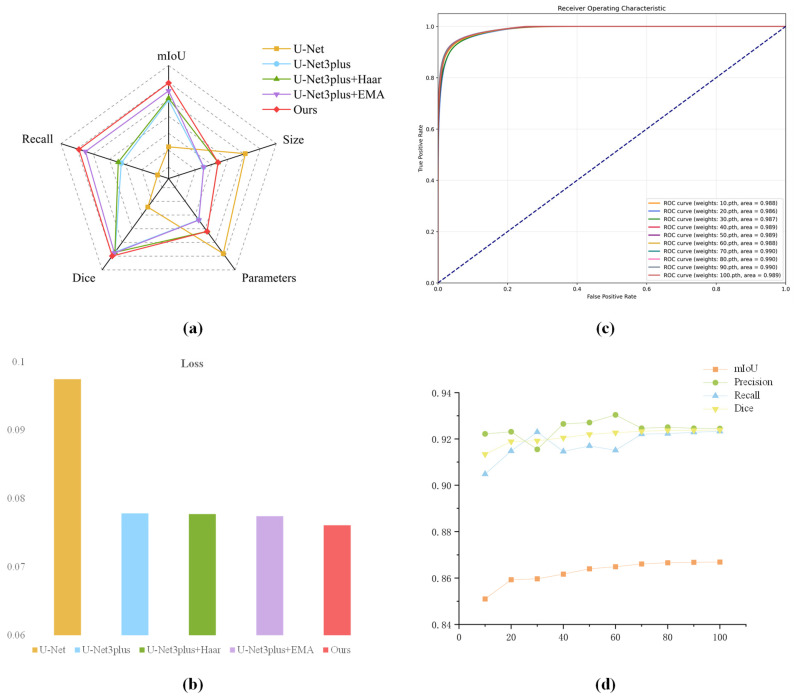
(**a**) Radar chart of model parameters. (**b**) Comparison of loss values across models using bar charts. (**c**) ROC curves for the Ours model weights at every 10-epoch interval. (**d**) Parameter change trends of the Ours model weights at every 10-epoch interval.

Figure 11a uses a radar chart to visually compare the segmentation capability and model size of five different models. Our model achieves the highest scores in mIoU, Recall, and Dice while maintaining relatively low model size and parameter count. Figure 11b, presented as a bar chart, shows that our model attains the lowest loss value, indicating smaller deviations from the ground truth and superior segmentation accuracy. As illustrated in Figure 11d, the metric values stabilize after 90 training epochs, which provides a reference for the number of training epochs in subsequent experiments. Within 100 epochs, the model converges stably, and the best-performing weights lie between epochs 90 and 100 (see Supplementary Material for related data). To further reduce parameter count and model size, future experiments will consider replacing the convolutional layers in the up-sampling module with depthwise separable convolutions.

### 3.3. Generalization Verification

#### 3.3.1. Mixed Dataset Performance Verification

To better evaluate the model’s generalization capability, the test set was subdivided into two main categories: diverse roots and various soils. Diverse roots include public datasets along with some scanner-acquired images. For the public dataset testing, we predicted peanut root systems captured via hyperspectral imaging, as well as root systems of various plants such as peanut, papaya, and cotton obtained from minirhizotron systems, in order to verify whether the model can segment roots from images acquired by different imaging methods. In addition, we predicted large-format images of complete cotton root systems (10,200 × 14,039 pixels) and complete wheat root systems (10,200 × 14,039 pixels) obtained by a scanner. These large images were split into smaller patches of 512 × 512 pixels, predicted separately, and then reassembled into the original large images. This process was used to verify whether the model can segment both taproot systems and fibrous root systems.

For the various soils category, the model predicted plant root images grown in different soil environments, including black soil, sandy soil, and ordinary soil, totaling 50 images. This was designed to test whether the model truly learns root features rather than relying on soil context. For further validation, the experiment categorized the images based on the degree of visual difference between roots and soil background. Images in which roots and background are clearly distinct, with sharp contour boundaries, are defined as Distinct. Images where the main root body is relatively distinguishable from the background but the root-soil boundaries appear similar are defined as Similar. Images where the root body closely resembles the soil background and the root contours are blurred are defined as Close.

**Figure 12:**
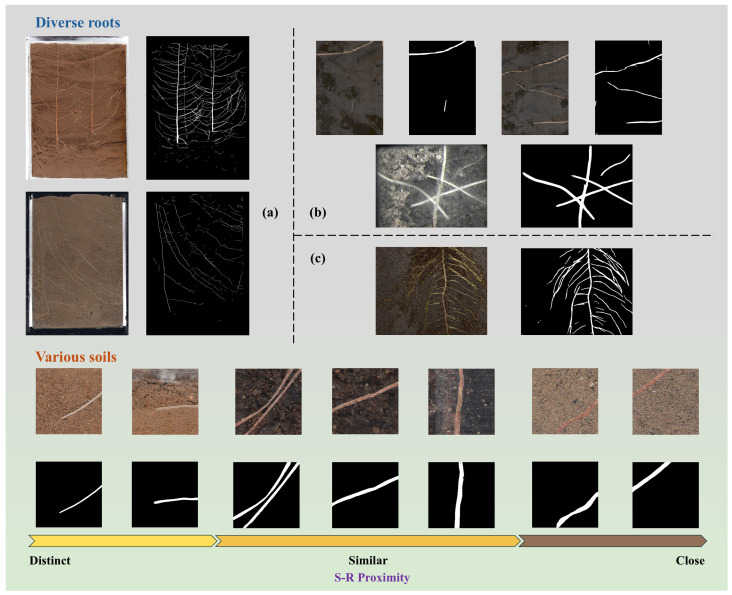
Generalization test of the model. (**a**) Complete cotton and wheat root system images and their segmentation results. (**b**) Root system images acquired via minirhizotron and their segmentation results. (**c**) Root system images acquired via hyperspectral imaging and their segmentation results.

As shown in Figure 12, our model is capable of segmenting two different types of plant root systems, namely taproot systems and fibrous root systems. The model performs effective segmentation on images acquired via hyperspectral imaging. However, its segmentation ability is poorer for images obtained through minirhizotron systems, as it struggles to capture fine details and is more susceptible to background interference, ultimately resulting in inferior segmentation outcomes.

After predicting plant root images in different soil environments, the root portions within the images can be effectively segmented by the model. Segmentation performance is better in ordinary soil and black soil. However, in sandy soil, roots are often occluded by soil particles, or root colors are similar to the soil background, resulting in low contrast, leading to poorer segmentation results. The model exhibits insufficient local segmentation accuracy for fine structures such as root tips and root hairs, with noticeable loss of edge details.

In response to the issues mentioned above, we considered optimizing the model through transfer learning. Previous studies have already validated the feasibility of transfer learning for root segmentation tasks [58]. Root images share similar characteristics; although differences exist in background complexity and morphological details of the root system, their core features are consistent. Therefore, to improve the model’s generalization capability, transfer learning offers certain advantages.

#### 3.3.2. Transfer Learning Enhances Model Generalization Performance

In this experiment, transfer learning was performed using a mixed training dataset that included cotton root images acquired by a scanner, as well as images of various plant roots, such as cotton and peanut, obtained via minirhizotron and hyperspectral methods, in order to enhance the model’s generalization capability. The best-performing weights from the initial training of our model were transferred. The backbone was frozen to preserve previously learned features, and to prevent the risk of overfitting, the number of epochs was reduced from 100 to 10. The best weights obtained after transfer learning were saved separately. For testing, the optimal weights were selected for prediction, and the evaluation metrics and inference results were compared before and after transfer learning using a mixed validation set of 3238 images.

Table 8 shows that after transfer learning, the model achieves significantly better overall scores on the multi-dataset test compared to the model before transfer learning, with mIoU increasing by 0.12% and Precision improving by 0.36%. The core of transfer learning lies in adapting the source domain to the target domain. Because the mixed dataset provides richer scenarios, noise patterns, and morphological variations, the model becomes more capable of distinguishing roots from the background, thereby improving metric values such as mIoU and Precision.

**Figure 13:**
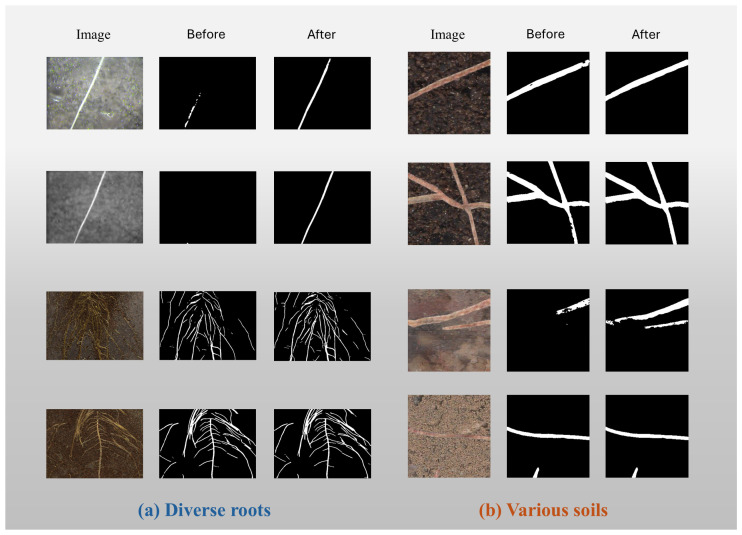
Comparison of the model before and after transfer learning. (**a**) Generalization on root-system images. (**b**) Generalization on soil-background images.

By comparing the network without transfer learning, we found that the transferred network can effectively segment plant root images acquired via minirhizotron systems, with segmentation performance clearly superior to that of the former. Transfer learning effectively mitigates issues such as occlusion by soil particles in minirhizotron images or blurred boundaries between roots and soil, substantially enhancing the model’s generalization capability. For hyperspectral images, segmentation performance was already good both before and after transfer learning; after transfer learning, the segmentation results improved somewhat, but the change was not pronounced (Figure 13a).

Figure 13b shows that after transfer learning, the model exhibits improved segmentation performance for root images in black-soil environments, while its segmentation of root images in ordinary-soil environments changes only marginally. Compared to the model without transfer learning, the transferred model demonstrates clearly better segmentation for root images in some complex sandy-soil environments. Because sandy soil has coarse particles and high permeability, and roots are similar in color to the light-colored soil, the model tends to misclassify roots as granular background in sandy-soil scenes. Therefore, achieving reliable root segmentation in sandy-soil scenarios represents an important breakthrough in model enhancement. The experiment revealed that the model’s segmentation of images acquired from different soil environments still has room for improvement. To optimize the model’s performance across diverse soil types, future work will consider collecting more complex and varied mixed datasets, enriching the diversity of data sources, and providing stronger data support for subsequent model refinement.

### 3.4. Onboard Testing

The experimental computer ran Windows 11. Docker was installed via the official website (https://docs.docker.com accessed on 25 June 2026) using the latest version of Docker Desktop. The Download for Windows installer was used, specifically version 27.5.1. WSL2 is a virtualization technology designed to provide Windows users with a highly integrated Linux runtime environment. The detailed deployment workflow is provided in Algorithm 1.
**Algorithm 1** WSL2 Configuration and Docker Container Deployment1: **Step 1: Environment Setup (WSL2)** 2: Open PowerShell as Administrator 3: Execute command: wsl--install 4: **Reboot** Computer to finalize installation 5: **Step 2: Docker Image Preparation** 6: Define Registry URL: *R* ← harbor.fsafsa.cn 7: Select Base Image: *I_base_* ← *R* +/public/customer/bingbinggan:v3 8: Encapsulate *Model_Env* and Code into Ibase 9: Target OS Version ← Ubuntu 20.04 10: **Step 3: Container Instantiation** and **Volume Mapping** 11: Define Host Path (Input): *P_host_in_* ← C:/YourInputFolder 12: Define Host Path (Output): *P_host_out_* ← C:/YourOutputFolder 13: **Create Container** C from Ibase with parameters: 14:     Mount *P_host_in_* → /img (Import) 15:     Mount *P_host_out_* ← /img_out (Export) 16: **Launch** Docker Desktop 17: **if** C is visible in Dashboard **then** 18:     Deployment Successful 19: **end if**

**Figure 14:**
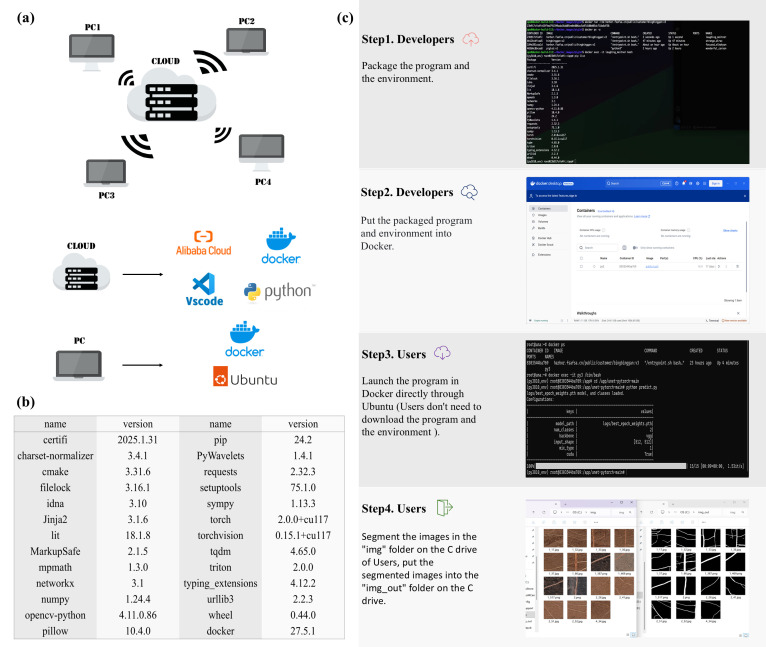
Process diagram of packaged environment versions and user-friendliness enhancement. (**a**) Schematic diagram of the deployment scheme. (**b**) Dependency packages and version information of the packaged environment. (**c**) Step-by-step operation workflow for developers and users.

To demonstrate that Docker enhances user-friendliness, we conducted an experiment on another Windows 11 computer without pre-installed programs or the required runtime environment. By launching and running the container in Docker Desktop, entering the container terminal in Ubuntu, and executing the fixed program command python predict.py, the images in the img folder on the C drive of this computer can be predicted, and the predicted images are saved back to the img_out folder on the C drive (Figure 14).

Deep-learning models typically depend on complex software environments, such as specific Python versions, deep-learning frameworks, and various dependency libraries. Using Docker, these environments can be packaged into a self-contained container, enabling researchers from other disciplines to quickly upload root images from their own computers and obtain recognition results through fixed, simple instructions—without manually writing code or debugging the environment. This approach ensures data privacy and security while saving considerable time and effort, lowering technical barriers across disciplines and accelerating progress in root-related research. In future work, we aim to streamline the operational steps further toward code-free operation, allowing image recognition to be performed through a web-based interface or dedicated software.

**Figure 15:**
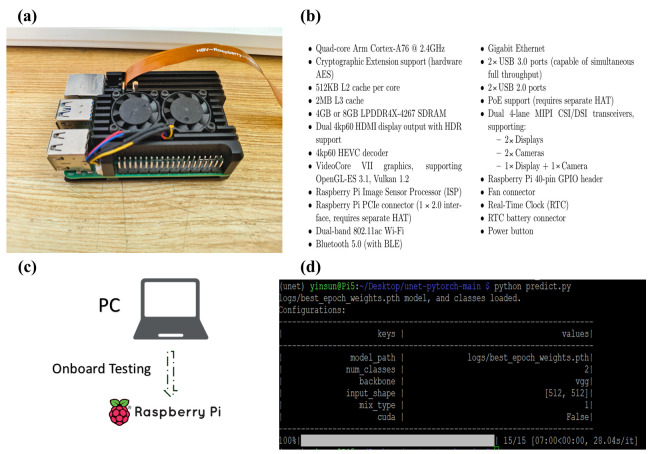
Raspberry Pi effectiveness testing. (**a**) Physical photograph of the Raspberry Pi used in the experiment. (**b**) List of hardware specifications of the Raspberry Pi. (**c**) Schematic diagram of the testing setup between the PC and the Raspberry Pi. (**d**) Terminal output of the model running on the Raspberry Pi.

In addition to evaluating performance using Docker, the experiment also conducted tests on a Raspberry Pi. The Raspberry Pi is a small single-board computer and was employed in this project as an edge device terminal. The specific model used was a Raspberry Pi 5 with 8 GB of RAM; relevant hardware and interface details are provided in Figure 15.

The experiment compared the single-image inference speed and total prediction time of our network when running the same set of images locally via the Docker interface, on a server, and on a Raspberry Pi. A total of 15 root images with a size of 512 × 512 pixels were predicted. When using Docker, the inference speed was approximately 1.53 it/s, resulting in a total prediction time of 9 s for the 15 images. On the server, the inference speed reached 2.69 it/s, with a total prediction time of 5 s. In contrast, the Raspberry Pi achieved a speed of only 0.036 it/s, requiring about 420 s to process the same 15 images.

The inference speed difference between Docker deployment and server deployment was relatively small. Moreover, server-side testing is prone to environment-configuration issues and is not readily accessible for ordinary developers. Docker deployment eliminates the need for manual installation of various dependencies on the local computer and avoids version conflicts that could lead to failures in wavelet-component extraction. Additionally, because the Raspberry Pi lacks GPU-acceleration support, its prediction efficiency is significantly lower compared to Docker and server setups. Nevertheless, the onboard deployment test on the Raspberry Pi demonstrates that our model can run stably on low-compute hardware, providing essential support for the feasibility of deploying the model in field-oriented, practical hardware scenarios.

## 4. Conclusions

This paper proposes a novel wavelet-enhanced full-scale segmentation network. The model adopts the full-scale skip-connection framework of U-Net3plus and integrates Haar wavelet downsampling and an EMA module. Wavelet transform replaces max-pooling as the downsampling operation, and the influence of wavelet transform on the network’s focus is visually validated through Grad-CAM. A multi-scale attention mechanism is introduced during the feature fusion stage, and the effectiveness of the EMA module is evaluated from the perspective of boundary accuracy using HD95. The segmentation performance is verified both subjectively through image assessment and objectively via quantitative metrics. Compared with the original U-Net, our model achieves a 1.13% higher mIoU, a 2.92% higher Recall, and a Dice score of 92.39%, while reducing both parameter count and model size and improving overall accuracy. Transfer learning is employed to enhance the model’s generalization capability, with tests conducted on root images from diverse roots and various soils. A Docker-based, simplified and secure root image segmentation interface is designed. Future work may explore the development of a website or standalone software to further enable code-free operation, making the tool more accessible to researchers from non-technical disciplines. In addition, on-board testing through deployment on edge devices provides hardware-level validation of the model’s practical deployment feasibility.

However, this study still has certain limitations. When roots are severely occluded by soil particles, the model still suffers from root breakage and cannot infer the root direction in the occluded regions. Furthermore, in extreme scenarios where the soil and root colors are extremely similar, the fine-root detection capability of the model still needs improvement. In future research, to address the root breakage problem caused by soil occlusion, we will leverage prior knowledge such as root continuity and branching patterns to complete the broken regions, thereby obtaining more complete root morphology. At the same time, we will consider model compression methods such as pruning and quantization to reduce model size and improve operational efficiency, so as to better adapt to the deployment requirements on edge devices.

## Figures and Tables

**Table 1:** Dataset split.

Acquisition Method	In Situ Root Segmentation	Transfer Learning
Ours (RhizoPot) 13,694 images	70% for training and 20% for validation 10% for testing	70% for training and 20% for validation 10% for testing
PRMI Dataset 2000 images	All for testing model generalization performance	70% for training and 20% for validation 10% for testing
HyperPRI Dataset 500 images	All for testing model generalization performance	70% for training and 20% for validation 10% for testing
Different Soil Conditions 50 images	All for testing model generalization performance	All for testing model generalization performance

**Table 2:** Hyperparameter configurations.

Hyperparameters	In Situ Root Segmentation	Transfer Learning
Batch size	2	2
Optimizer	Adam	Adam
Initial Max Learning Rate	0.0001	0.0001
Min Learning Rate	0.000001	0.000001
epoch	100	10
Frozen Training	False	Backbone Frozen
stride	1	1
bilinear	True	True
Input Image Channels	3	3

**Table 3:** Performance comparison of base segmentation networks.

							Model Size	
Model	mIoU (%)	Precision (%)	Recall (%)	Dice (%)	IoU (%)	Loss (%)	Parameters (M)	Memory (MB)
U-Net	85.56	91.01	89.41	90.25	82.23	9.75	31.03	118.48
U-Net2plus	86.34	93.41	**90.97**	92.17	85.48	7.83	47.17	180.04
U-Net3plus	**86.39**	**93.73**	90.76	**92.22**	**85.57**	**7.78**	**26.97**	**103.01**
TransUNet	84.80	91.07	90.17	90.62	82.85	9.38	105.91	404.19
SwinUNet	67.09	88.09	69.92	77.96	63.88	22.04	27.16	103.64

Bold values indicate the best results among all comparison models for each evaluation metric.

**Table 4:** Supplementary comparison with other segmentation architectures.

Model	IoU (%)	Precision (%)	Recall (%)	Dice (%)
UKAN	72.71%	84.67	83.74	84.20
Mamba U-Net	Failed	-	-	-
DeepLabV3+	81.59	89.49	90.24	89.86
CBAM-UNet	84.49	93.06	90.17	91.59

**Table 5:** Comparison of different wavelet transform models.

Model	Haar	DB2	Inverse Transform	mIoU (%)	Precision (%)	Recall (%)	Dice (%)
Model1				86.39	**93.73**	90.76%	92.22%
Model2	√			**86.42**	93.64	**90.87%**	**92.23%**
Model3		√		86.17	92.90	90.23%	91.55%
Model4	√		√	86.25	93.02	90.48%	91.73%

Bold values indicate the best results among all comparison models for each evaluation metric. A checkmark (√) indicates that the model has adopted the corresponding module.

**Table 6:** Evaluation of attention mechanisms.

Model	mIoU (%)	Precision (%)	Recall (%)	Dice (%)	IoU (%)	Loss (%)	HD95 (px)
U-Net3plus + Haar	86.42	**93.64**	90.87	92.23	85.59	7.77	5.15
CBAM	86.51	92.65	91.90	92.27	85.66	7.73	5.16
EMA	**86.69**	92.45	**92.33**	**92.39**	**85.86**	**7.61**	**4.48**

Bold values indicate the best results among all comparison models for each evaluation metric.

**Table 7:** Evaluation metrics for various improved models.

Method	mIoU (%)	Precision (%)	Recall (%)	Dice (%)	IoU (%)	Loss (%)	FLOPS(G)	Inference Speed (it/s)
Baseline	86.69 ± 0.37	93.73 ± 0.14	90.73 ± 0.20	92.20 ± 0.14	85.54 ± 0.24	7.80 ± 0.14	**799.73**	**5.60**
Baseline + EMA	86.80 ± 0.36	92.91 ± 0.5	91.99 ± 0.56	92.42 ± 0.25	85.96 ± 0.44	7.55 ± 0.25	810.20	5.07 it/s
Baseline + Haar	86.71 ± 0.38	**94.12 ± 0.58**	90.79 ± 0.24	92.45 ± 0.24	85.92 ± 0.41	7.58 ± 0.24	804.05	5.48 it/s
WaveUNet+ (Baseline + Haar + EMA)	**87.08 ± 0.41**	92.94 ± 0.56	**92.34 ± 0.24**	**92.64 ± 0.27**	**86.29 ± 0.46**	**7.36 ± 0.27**	814.53	5.00 it/s

Bold values indicate the best results among all comparison models for each evaluation metric.

**Table 8:** Comparison of evaluation metrics before and after transfer learning.

Model	mIoU (%)	Precision (%)	Recall (%)	Dice (%)	IoU (%)	Loss (%)	F1 (%)
Before	86.78	92.87	**92.04**	92.45	85.97	7.55	92.45
After	**86.90**	**93.23**	91.85	**92.53**	**86.11**	**7.47**	**92.53**

Bold values indicate the best results among all comparison models for each evaluation metric.

## Data Availability

The data are available in a publicly accessible repository. The code can be obtained from https://github.com/WLL-cyber/WaveUNet-.git (accessed on 16 June 2026).

## Supplementary Materials

The following supporting information can be downloaded at: https://www.mdpi.com/article/10.3390/plants15132034/s1, Figure S1: Comparison of layer-wise Grad-CAM heatmaps at different training epochs; Table S1: Detailed parameter information of each model and confidence intervals of the final model.
