# Supplementary figures and images for: WaveUNet+: Preserving Root System Architecture Integrity in In Situ Root Segmentation via a Unified Spectral–Spatial Framework
Source: Plants (Basel). 2026 Jun 30;15(13):2034. doi: 10.3390/plants15132034 (PMC13364121; doi:10.3390/plants15132034)

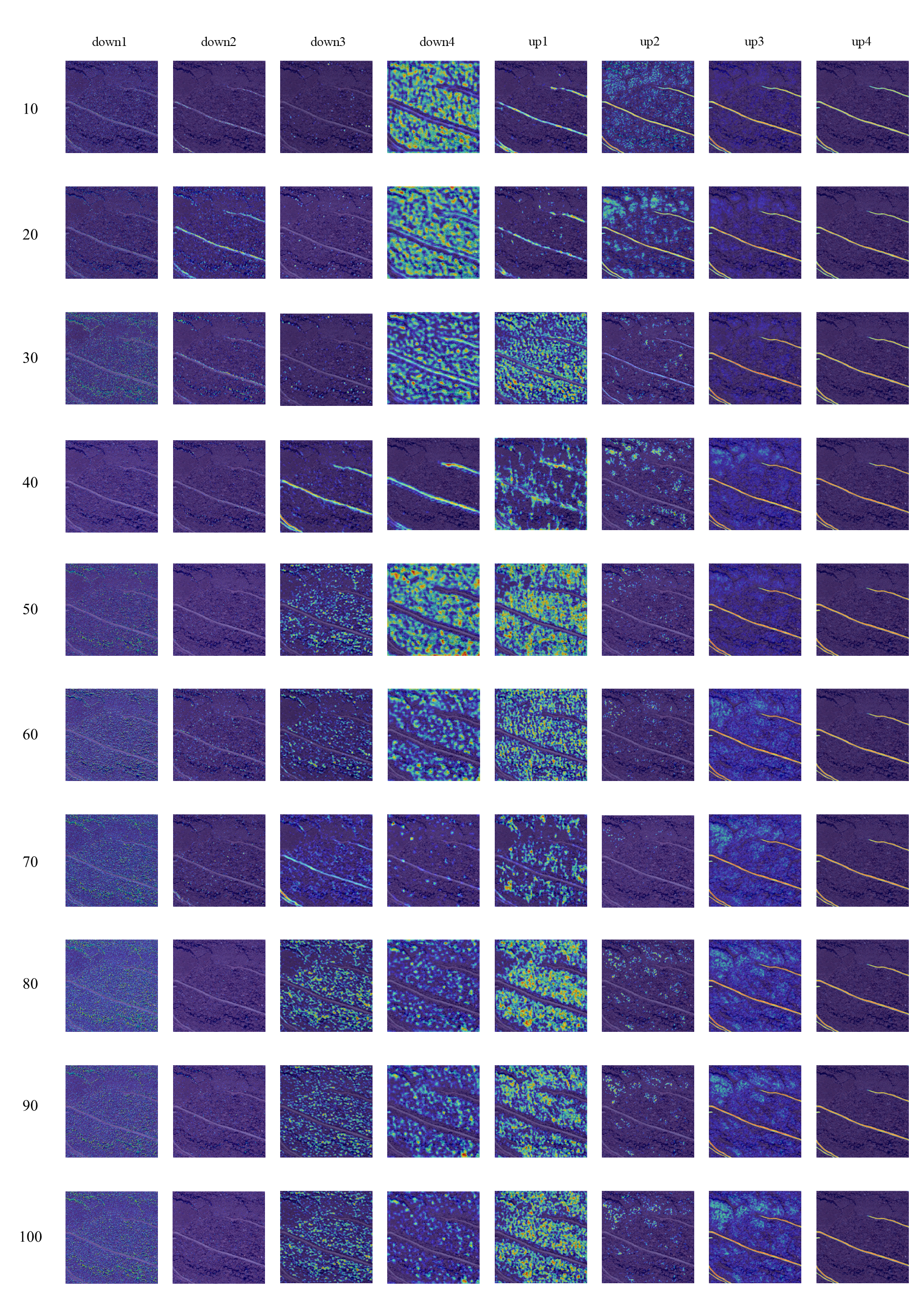

Supplement: Supplementary file 1 [file plants-15-02034-s001.zip › Supplementary Material-Figure S1.tif]
